# MoO_3_ on zeolites MCM-22, MCM-56 and 2D-MFI as catalysts for 1-octene metathesis

**DOI:** 10.3762/bjoc.14.272

**Published:** 2018-11-27

**Authors:** Hynek Balcar, Martin Kubů, Naděžda Žilková, Mariya Shamzhy

**Affiliations:** 1 J. Heyrovský Institute of Physical Chemistry of the Czech Academy of Sciences, v.v.i., Dolejškova 3, 182 23 Prague 8, Czech Republic

**Keywords:** metathesis, molybdenum oxide, 1-octene, thermal spreading, zeolites

## Abstract

Highly active olefin metathesis catalysts were prepared by thermal spreading MoO_3_ and/or MoO_2_(acac)_2_ on MWW zeolites (MCM-22, delaminated MCM-56) and on two-dimensional MFI (all in NH_4_^+^ form). The catalysts‘ activities were tested in the metathesis of neat 1-octene (as an example of a longer chain olefin) at 40 °C. Catalysts with 6 wt % or 5 wt % of Mo were used. The acidic character of the supports had an important effect on both the catalyst activity and selectivity. The catalyst activity increases in the order 6MoO_3_/HZSM-5(25) (Si/Al = 25) << 6MoO_2_(acac)_2_/MCM-22(70) < 6MoO_3_/2D-MFI(26) < 6MoO_3_/MCM-56(13) < 6MoO_3_/MCM-22(28) reflecting both the enhancing effect of the supports‘ acidity and accessibility of the catalytic species on the surface. On the other hand the supports‘ acidity decreases the selectivity to the main metathesis product C14 due to an acid-catalyzed double bond isomerization (followed by cross metathesis) and oligomerization. 6MoO_3_/2D-MFI(26) with a lower concentration of the acidic centres resulting in catalysts of moderate activity but with the highest selectivity.

## Introduction

Molybdenum oxide on silica, alumina or silica-alumina belongs to the well-known and long-time used metathesis catalysts [1]. Albeit typical ill-defined catalysts they are still popular as relatively cheap catalysts finding industrial applications especially in the treatment of low olefins [2–5]. Their catalytic activity depends on many factors, especially on Mo loading, support acidity, and pre-reaction activations. Surface isolated MoO_4_ tetrahedra were proved as the main precursors of the catalytic species [6–7], thus the perfect dispersion of MoO_3_ on the surface is a crucial precondition for a high catalytic activity. The mechanisms of transformation of these precursors to the surface Mo carbenes as real catalytically active species has been suggested [6–7]. The replacement of ordinary silicas for mesoporous molecular sieves SBA-15 or MCM-41 increased the catalyst activity substantially, which allowed performing the metathesis of long chain olefins under mild reaction conditions [8–10]. The positive effect of these supports on the catalyst activity was ascribed to their high surface areas enhancing the spreading of MoO_3_ molecules on the surface and large pores increasing the substrates/products transport rate.

**Scheme 1:**
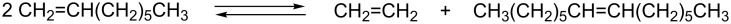
1-Octene metathesis reaction.

Microporous zeolites like HZSM-5 impregnated by ammonium heptamolybdate solutions were used for the metathesis of low olefins (ethylene, propylene, butenes) [11–13]. In the case of bulkier substrates they suffer, however, of micropore size limitations. To overcome these limitations a decrease in crystal size and the application of two-dimensional zeolites can be used [14–17]. Two dimensional 2D-MFI and MWW delaminated zeolite MCM-56, which have been prepared recently [18–21], represent two types of these materials, which exhibit relatively high surface areas and high accessibility of catalytic sites on the surface as well [22]. Therefore, we supported MoO_3_ and/or MoO_2_(acac)_2_ on (i) 2D-MFI (and ordinary HZSM-5 for comparison) and similarly on (ii) MCM-56 and its 3D analogue MCM-22 (both in NH_4_^+^ form) and examined their activity in the metathesis of neat 1-octene (Scheme 1) under ambient pressure and 40 °C. According to our best knowledge, none of these materials have been tested as supports for MoO_3_ based catalysts for metathesis of higher alkenes up to now. MoO*_x_* on MCM-22 combined with γ-Al_2_O_3_ was used in cross metathesis of 2-butene and ethylene in a stream (125 °C, 1 MPa) [23]. MCM-22, and MCM-56 were also used as supports for Hoveyda–Grubbs type hybrid catalysts active in metathesis of long-chain unsaturated esters [24].

## Results and Discussion

### Catalyst preparation and characterization

**Figure 1:**
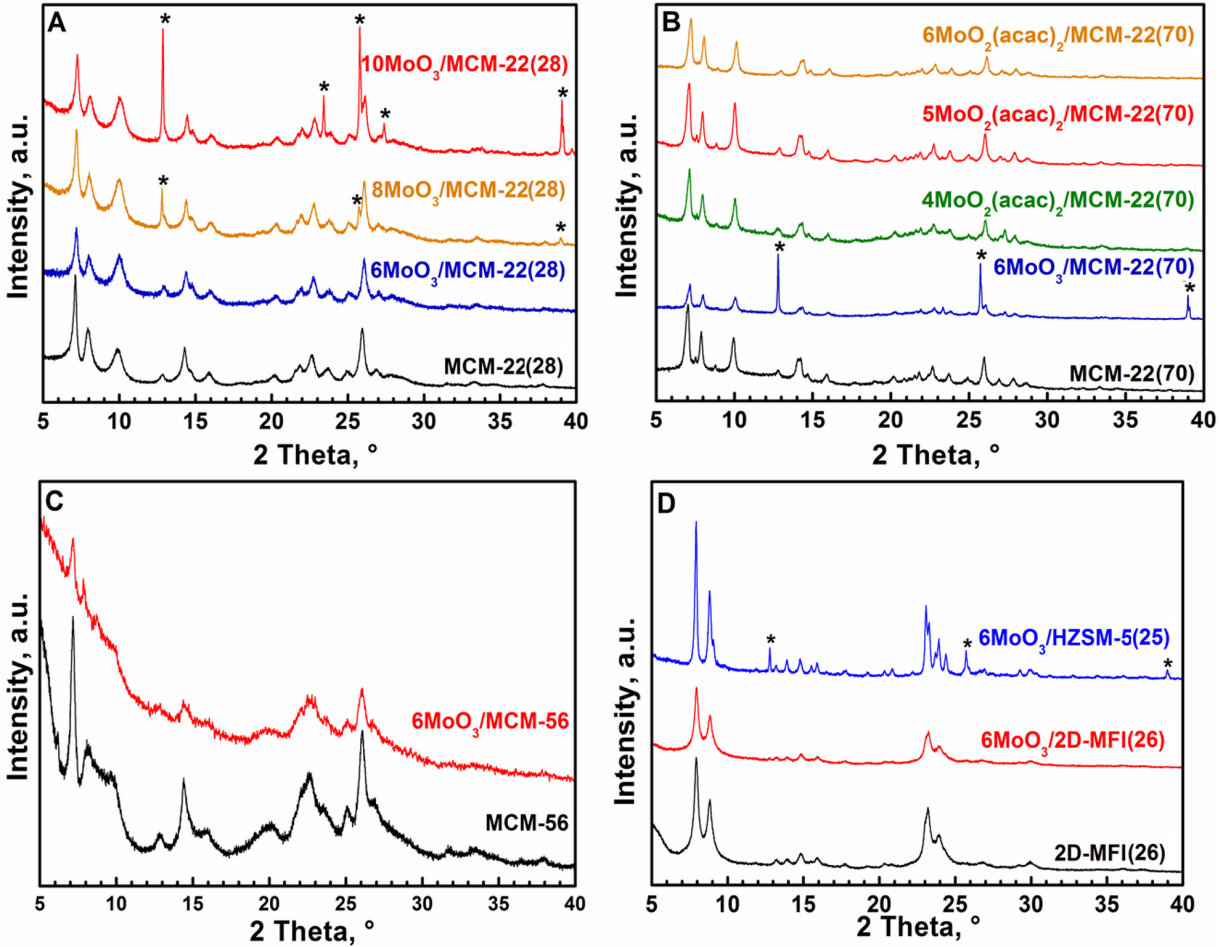
A,B,C,D: XRD patterns of parent supports and catalysts used. Asterisk marks MoO_3_.

XRD patterns and texture properties (Table 1, Figure 1 A,B,C,D) of prepared MCM-22, MCM-56 and 2D-MFI zeolites proved a high quality of these supports. For catalyst labelling following the mode has been adopted: *x* MoO_3_/MCM-22(*y*), where *x* = Mo concentration in wt % Mo, *y* = Si/Al molar ratio. After spreading Mo compounds over the support surface areas (*S*_BET_, *S*_ext_) as well as void volumes (*V*) decreased. Similar reduction of these quantities has been already observed earlier [9–10,24]. For *x* MoO_3_/MCM-22(28), XRD patterns of catalysts are similar to those of their parents approximately up to *x* = 6 wt % of Mo (0.9 Mo atoms per nm^2^). At higher Mo concentrations signals of crystalline MoO_3_ appeared (marked with * in Figure 1 A,B,D). It suggests 6 wt % of Mo being the optimal Mo loading. On the other hand, *x* MoO_3_/MCM-22(70) catalyst with *x* = 6 wt % Mo exhibited slight MoO_3_ signals, when prepared from MoO_3_ probably due to the lower surface area (especially external one) comparing with MCM-22(28). However, when MoO_2_(acac)_2_ was used as a source of Mo, catalysts with 6 wt % (and lower) content of Mo did not exhibit any MoO_3_ signals. It is consistent with the previous observation that MoO_2_(acac)_2_ provided better catalyst than MoO_3_ [9]. XRD patterns of 6MoO_3_/MCM-56(13) and 6MoO_3_/2D-MFI(26) indicated also a good MoO_3_ spreading, contrary to 6MoO_3_/HZSM-5(25) where MoO_3_ signals were clearly visible, probably as a result of lower external surface area.

**Table 1:** Texture properties of catalysts and corresponding supports.

	catalyst	*S*_BET_ (m^2^/g)	*S*_ext_ (m^2^/g)	*V* (cm^3^/g)

1	MCM-22(28)	455	119	0.59
2	6MoO_3_/MCM-22(28)	423	119	0.38
3	6MoO_2_(acac)_2_/MCM-22(28)	426	94	0.57
4	MCM-22(70)	421	58	0.29
5	6MoO_3_/MCM-22(70)	180	39	0.25
6	6MoO_2_(acac)_2_/MCM-22(70)	355	41	0.24
7	2D-MFI(26)	565	343	0.61
8	6MoO_3_/2D-MFI(26)	478	221	0.57
9	MCM-56(13)	469	164	0.57
10	6MoO3/MCM-56(13)	269	129	0.55
11	HZSM-5(25)	410	44	0.23
12	6MoO3/HZSM-5(25)	388	38	0.23

*S*_BET_ = BET area, *V* = total void volume (*p*/*p*_0_ = 0.95), *S*_ext_ = external surface (from *t*-plot).

Contrary to the all-siliceous mesoporous sieves (like SBA-15) which are neutral, zeolites are acidic and their acidity (both Brønsted and Lewis-type) plays an important role for catalysis. The acid site concentrations of zeolitic supports and the corresponding catalysts measured using FTIR spectroscopy of adsorbed pyridine are shown in Table 2, while the relevant IR spectra are shown in Supporting Information File 1 (Figures S1–S5). It is seen that all supports contained both Brønsted and Lewis acid sites of various strength. MCM-22(28) and MCM-56(13) exhibited the highest concentrations of acid sites (both Brønsted and Lewis) in accord with their highest Al concentrations. The acid sites concentrations of MCM-22(70) and 2D-MFI(26) were lower and close to each other. The Brønsted acid site concentration of HZSM-5(25) was as high as that of MCM-22(28), however, its Lewis acid site concentration was significantly lower. After supporting Mo compounds the concentrations of Brønsted acid sites decreased significantly which may indicate that MoO*_x_* species reacted predominantly with Brønsted acid sites of the supports. It is manifested by intensity decrease of the band in the region 3609–3625 cm^−1^, ascribed to OH vibration in the Si–O(H)–Al acid site (see Supporting Information File 1, Figures S1–S5) [25]. On the other hand, the concentrations of Lewis acid sites in the catalysts was slightly higher compared to the parent supports. It may be explained by the formation of some amount of Mo in a lower oxidation state which has been already described for siliceous supports (MCM-41, SBA-15) [9,26].

**Table 2:** Acid site concentrations in catalysts and corresponding supports.^a^

sample	*c*(B)^b^, mmol/g				*c*(L)^c^, mmol/g			

	150 °C	250 °C	350 °C	450 °C	150 °C	250 °C	350 °C	450 °C
	
MCM-22(70)	0.11	0.12	0.09	0.02	0.06	0.04	0.03	0.03
6MoO_3_/MCM-22(70)	0.02	0.01	–	–	0.10	0.01	0.01	–
6MoO_2_(acac)_2_/MCM-22(70)	0.06	0.06	0.05	0.02	0.16	0.07	0.04	0.03
MCM-22(28)	0.29	0.32	0.30	0.19	0.13	0.09	0.08	0.06
6MoO_3_/MCM-22(28)	0.07	0.09	0.07	0.04	0.31	0.10	0.03	0.01
6MoO_2_(acac)_2_/ MCM-22(28)	0.09	0.07	0.05	0.02	0.23	0.08	0.03	0.01
2D-MFI(26)	0.13	0.13	0.08	0.02	0.07	0.07	0.06	0.04
6MoO_3_/2D-MFI(26)	0.06	0.04	0.03	0.01	0.10	0.03	0.01	–
HZSM-5(25)	0.29	0.28	0.22	0.07	0.04	0.03	0.02	0.02
6MoO_3_/HZSM-5(25)	0.14	0.12	0.09	0.03	0.17	0.07	0.04	0.04
MCM-56(13)	0.23	0.17	0.13	0.04	0.18	0.12	0.09	0.08
MoO_3_/MCM-56(13)	0.04	0.03	0.01	–	0.17	0.06	0.03	0.02

^a^Determined by FTIR. ^b^Brønsted acid site. ^c^Lewis acid site.

### Catalytic activity

#### MCM-22-based catalysts

Na^+^ forms of zeolites turned out to be unsuitable supports for metathesis catalysts. For example, by supporting MoO_3_ on MCM-22(28) in Na^+^ form (6 wt % of Mo) we obtained material providing only 0.5% 1-octene conversion in 19 h (1-octene/Mo = 320, *t* = 40 °C). Therefore, we converted Na^+^ forms to NH_4_^+^ forms, which were used for supporting Mo compounds by thermal spreading method.

**Figure 2:**
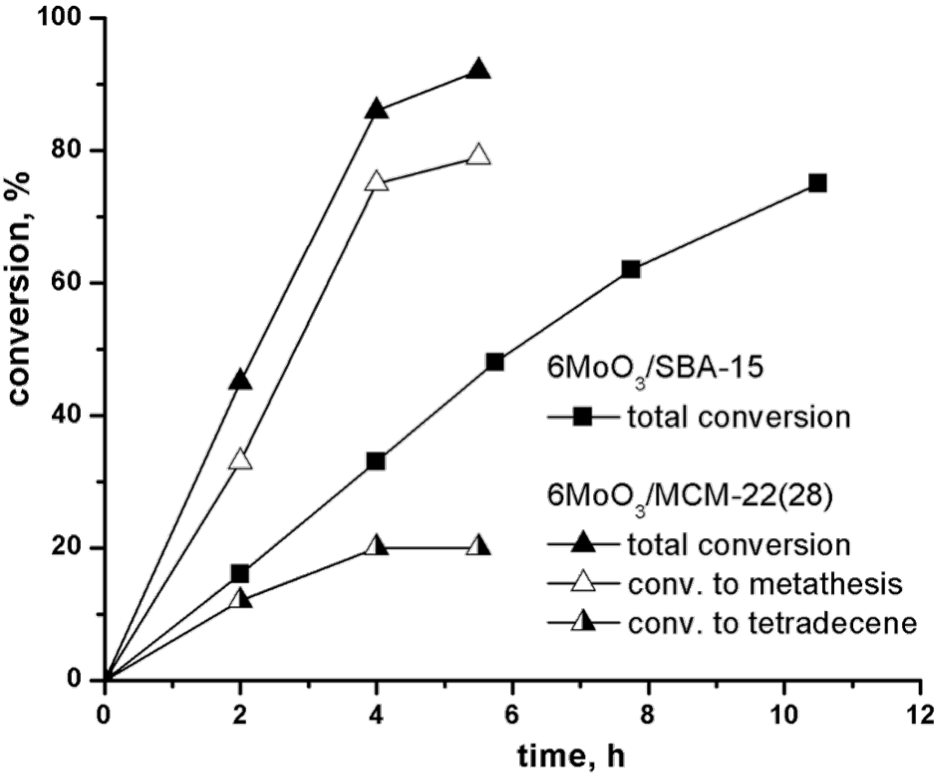
Conversion vs time curves for 1-octene metathesis over 6MoO_3_/MCM-22(28) and 6MoO_3_/SBA-15. Neat 1-octene, 1-octene/Mo = 320, *t* = 40 °C.

The time development of 1-octene conversion over 6MoO_3_/MCM-22(28) is shown in Figure 2. The GC chromatogram of the final product is shown in Figure S6 (in Supporting Information File 1). It is seen that in addition to the main metathesis product (7-tetradecene), alkenes from C13 to C9 are present in considerable amounts. It is a consequence of the 1-octene double bond isomerization followed by cross metathesis. Moreover, a certain amount of oligomers (mainly dimers) were also observed in the reaction mixtures. Both isomerization and oligomerization are due to the acidic character of the support (vide infra). In addition to the total conversion of 1-octene (*K*_tot_), the conversion to all metathesis products (*K*_met_), and the conversion to tetradecene (*K*_C14_) calculated according to the following equations are plotted in Figure 2.


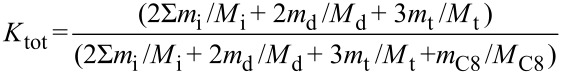



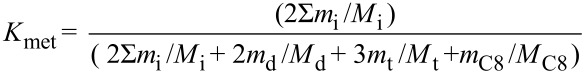



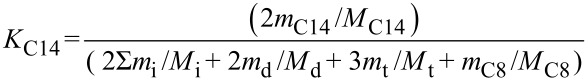


where *m*_i_ and *M*_i_ (i = 9–14) are weight amounts and molecular weights of alkenes from C9 to C14; *m*_d_, *m*_t_ and *M*_d_, *M*_t_ are weight amounts and molecular weights of octene dimers and trimers, respectively; *m*_C8_ is weight amount of octene (all isomers) and *M*_C8_ is the molecular weight of octene.

For comparison, the conversion curve over 6MoO_3_/SBA-15 is added in Figure 2. 6MoO_3_/SBA-15 was prepared from all-siliceous SBA-15 (*S*_BET_ = 877m^2^/g, *V* = 1.07cm^3^/g, pore diameter *D* = 6.4 nm) and it is known as a very active and selective catalyst [9–10]. Under reaction conditions applied the selectivity to tetradecene was about 98% during the whole experiment, and therefore only *K*_tot_ is plotted in Figure 2 in this case.

Both *K*_tot_ and *K*_met_ for 6MoO_3_/MCM-22(28) were significantly higher than the total conversion for 6MoO_3_/SBA-15 (Figure 2). Conversions to oligomers (*K*_ol_ = *K*_tot_ − *K*_met_) were about 12% (at 2 h) and practically did not change in the further course of the reaction. However, the conversions to tetradecene were rather low (maximum conversion about 20% was achieved). Higher catalytic activity of molybdenum oxide on zeolitic support in metathesis may be ascribed to the higher acidity of supports. The enhancing effect of Brønsted acidity on the catalytic activity has been already described [6] and it assumed that most of Mo active species in zeolite-based catalysts are formed by reacting molybdenum oxide with Si-O(H)-Al groups [12,27]. Similarly, Lim et al. showed recently [28], that Brønsted acid sites improve dispersion of molybdenum oxide on the surface. Moreover, for related system based on tungsten oxide in zeolite, it was suggested using high resolution STEM that Brønsted acid sites in proximity to metathesis active sites facilitate olefin adsorption and metallocycle formation [29]. Such mechanism may be effective also for Mo catalysts. The decrease in the selectivity due to isomerization and/or oligomerization seems to be an unavoidable cost for this activity enhancement.

It is known for molybdenum oxide catalysts, that with increasing Mo loading the catalytic activities increase up to maximum value [6,10]. At higher loadings the molybdenum oxide spreading on the surface became imperfect and catalytically inactive bulk MoO_3_ appears. The effect of increasing Mo loading on catalyst activity for MCM-22(28)-based catalyst is shown in Table 3.

**Table 3:** The effect of Mo loading on catalyst activity in 1-octene metathesis.^a^

catalyst	reaction time, h	*K*_tot_, %	*K*_met_, %	*K*_ol_, %	*K*_C14_, %

6MoO_3_/MCM-22(28)	2 4 6	45 86 92	33 75 79	12 11 13	12 20 20
8MoO_3_/MCM-22(28)	2 4 6.5 22	21 41 58 85	15 35 51 77	6 6 7 8	10 18 25 36
10MoO_3_/MCM-22(28)	2 4 6	2.6 3.4 4	0.6 0.7 1	2 2.7 3	0.6 0.7 1

^a^50 mg Catalyst, 1.5 mL 1-octene, 40 °C.

For 8MoO_3_/MCM-22(28) XRD pattern shows a small amount of bulk MoO_3_ (marked with asterisk in Figure 1A). In accord with this, the conversions fell down in comparison with 6MoO_3_/MCM-22(28), the selectivity, however, slightly increased: the amount of oligomers was reduced and the selectivity to the tetradecene approximately doubled. It suggests that more acid sites were covered by MoO*_x_* species and oligomerization and isomerization ability of catalysts decreased. However, further increase in the Mo loading to 10 wt % in 10MoO_3_/MCM-22(28) led nearly to the lost of catalytic activity, which is explained by deposition of Mo in the catalytically inactive bulk MoO_3_. Correspondingly, very intensive diffraction lines of the bulk MoO_3_ appeared in the XRD pattern of 10MoO_3_/MCM-22(28) (see Figure 1A).

To reduce isomerization and oligomerization ability of MCM-22-based catalysts we prepared zeolite with Si/Al = 70 (and therefore with lower acidity – vide supra): MCM-22(70). The results showing the catalytic behavior of the prepared MCM-22(70)-based catalysts 6MoO_2_(acac)_2_/MCM-22(70), 5MoO_2_(acac)_2_/MCM-22(70), and 4MoO_2_(acac)_2_/MCM-22(70) are collected in Table 4.

**Table 4:** 1-Octene metathesis over MCM-22(70)-based catalysts.^a^

catalyst	reaction time, h	*K*_tot_, %	*K*_met_, %	*K*_ol_, %	*K*_C14_, %

6MoO_3_/MCM-22(70)	2 4.5 6	2 3 2	–	–	–
6MoO_2_(acac)_2_/MCM-22(70)	2 4 22	8 9 11.5	7.5 8 10.5	0.5 1 1	5 6 7
5MoO_2_(acac)_2_/MCM-22(70)	2.3 3.3 20	8 11 35	7 10 32	1 1 3	5 7 17
4MoO_2_(acac)_2_/MCM-22(70)	2 4 21	11 16 16	11 15 15	0 1 1	10 14 14

^a^50 mg Catalyst, 1.5 mL 1-octene, 40 °C.

XRD pattern of 6MoO_3_/MCM-22(70) exhibited some amount of bulk MoO_3_ (Figure 1B). Evidently on this less acidic support the MoO_3_ spreading is not perfect, which explains its negligible activity in metathesis reaction. However, using bis(acetylacetonate) complex MoO_2_(acac)_2_ as a source of Mo we obtained 6MoO_2_(acac)_2_/MCM-22(70), 5MoO_2_(acac)_2_/MCM-22(70), and 4MoO_2_(acac)_2_/MCM-22(70) exhibiting no signals of bulk MoO_3_ in XRD pattern (Figure 1B) and showing a mild metathesis activity. The highest conversion *K*_tot_ = 35% (after 20 h) was achieved over 5MoO_2_(acac)_2_/MCM-22(70). Oligomerization activity of all these catalysts was considerably lower in comparison with that of 6MoO_3_/MCM-22(28) (*K*_ol_ = 1% only). However, the isomerization was not suppressed and conversion to tetradecene *K*_C14_ was low.

#### MCM-56-based catalysts

**Figure 3:**
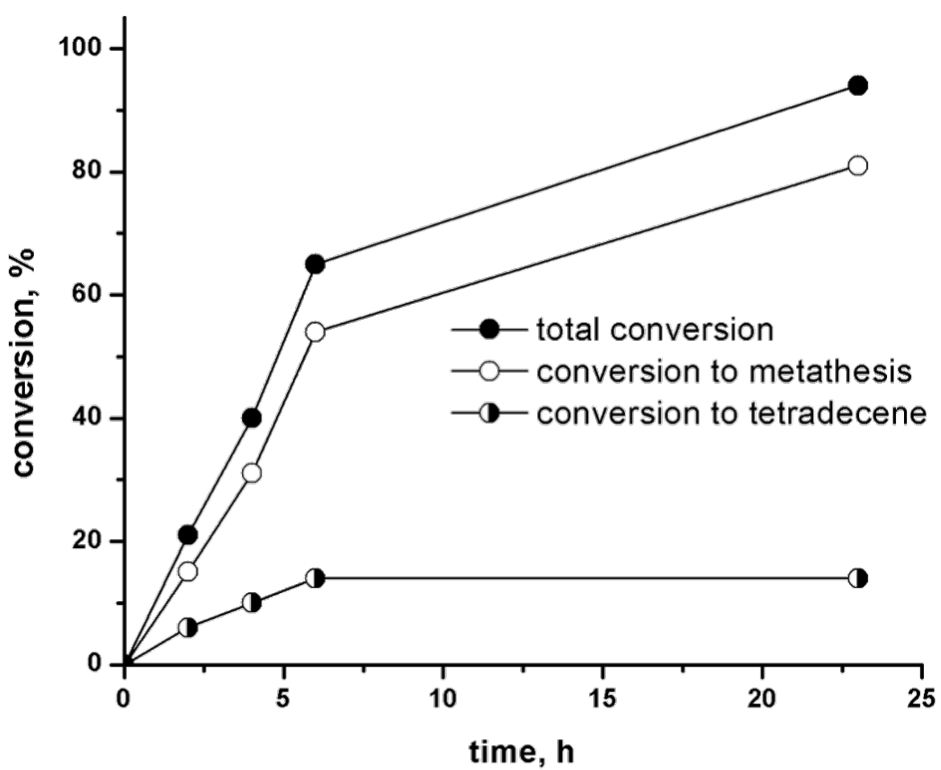
Conversion vs time curves for the 1-octene metathesis over 6MoO_3_/MCM-56(13). Neat 1-octene, 1-octene/Mo = 320, *t* = 40 °C.

Conversion curves for the 1-octene metathesis over 6MoO_3_/MCM-56(13) under standard conditions are displayed in Figure 3. In spite of the 2D character of support the conversions over 6MoO_3_/MCM-56(13) were significantly lower in comparison with 6MoO_3_/MCM-22(28): the initial reaction rate (calculated at reaction time = 2 h) being about a half of the initial reaction rate over 6MoO_3_/MCM-22(28). On the other hand the extent of oligomerization was practically the same (for final product the oligomerization selectivity was 14%) and the extent of cross metathesis was even higher (the selectivity to tetradecene was only 15%). The crystals of MCM-22 (see SEM image in Supporting Information File 1, Figure S7) consist of very thin platelets and therefore a great amounts of 12-membered ring cups of MWW structure are on crystal exterior [18]. These cups as we assume host MoO*_x_* species. Although MCM-56(13) as 2D zeolite consists of very thin layers, these layers may be curled and packed, which prevents the access of substrate molecules to the most of 12MR cups (for MCM-56(13) morphology see Supporting Information File 1, Figure S8). This may explain the lower activity of 6MoO_3_/MCM-56(13) compared with 6MoO_3_/MCM-22(28). Similarly, a higher activity of MCM-22 in comparison with MCM-56 has been observed in toluene disproportionation [18] and also for RCM of citronellene over immobilized Ru catalysts the activity of catalyst based on MCM-56 was not higher than that based on MCM-22 [24].

#### MFI-based catalysts

**Figure 4:**
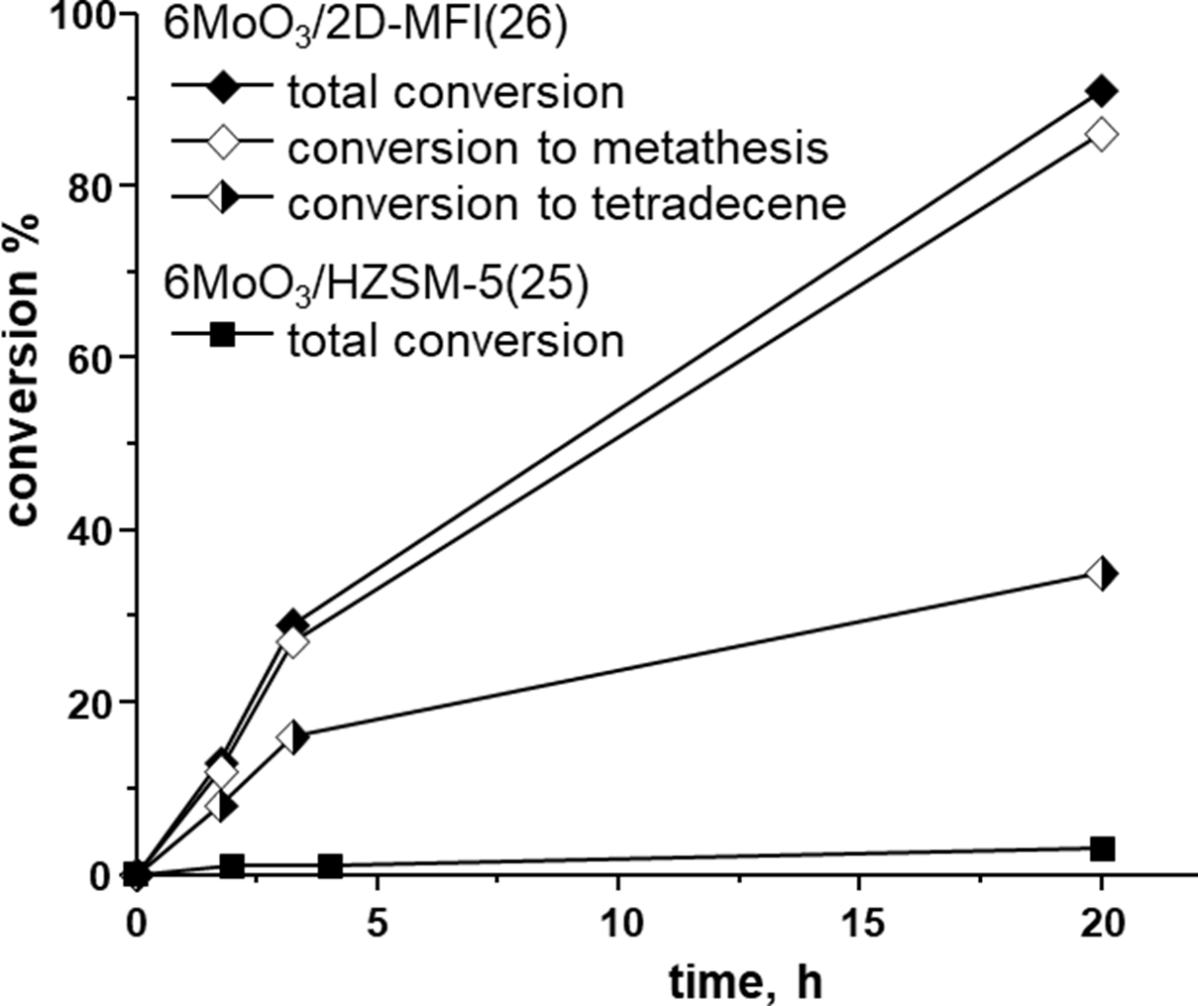
Conversion vs time curves for 1-octene metathesis over 6MoO_3_/2D-MFI(26) and 6MoO3/HZSM-5(25). Neat 1-octene, 1-octene/Mo = 320, *t* = 40 °C.

The comparison of conversion curves for 1-octene over 6MoO_3_/2D-MFI(26) and 6MoO_3_/HZSM-5(25) under standard conditions is given in Figure 4. It is seen that 6MoO_3_/HZSM-5(25) exhibited only negligible activity (*K*_tot_ = *K*_met_ = 3% after 20 h) in accord with poor MoO_3_ spreading (see Figure 1D). Despite the high acidity of the support, a poor accessibility of relevant surface OH groups during the thermal spreading process and a poor accessibility of possible active sites by substrate molecule during metathesis may cause 6MoO_3_/HZSM-5(25) to be practically inactive. On the other hand, over 6MoO_3_/2D-MFI(26) about 90% conversion was achieved for the same reaction time (20 h). The initial reaction rate over 6MoO_3_/2D-MFI(26) was only slightly lower than that over 6MoO_3_/MCM-56(13) and about one half of that over 6MoO_3_/MCM-22(28). Contrary to 6MoO_3_/MCM-22(28) the oligomerization activity of 6MoO_3_/2D-MFI(26) was reduced (*K*_ol_ was from 1% to 5%) and the selectivity to tetradecene was higher (for final conversions *K*_C14_/*K*_met_ = 0.41 and 0.25 for 6MoO_3_/2D-MFI(28) and 6MoO_3_/MCM-22(28), respectively). Lower acidity of 6MoO_3_/2D-MFI(28) may explain the lower extent of oligomerization and isomerization reactions and increased tetradecene selectivity. Lower acidity may also bring about the reduced activity as compared with 6MoO_3_/MCM-22(28); however, different structures of MCM-22 and MFI do not allow simple comparison.

### The accompanying oligomerization activity

**Figure 5:**
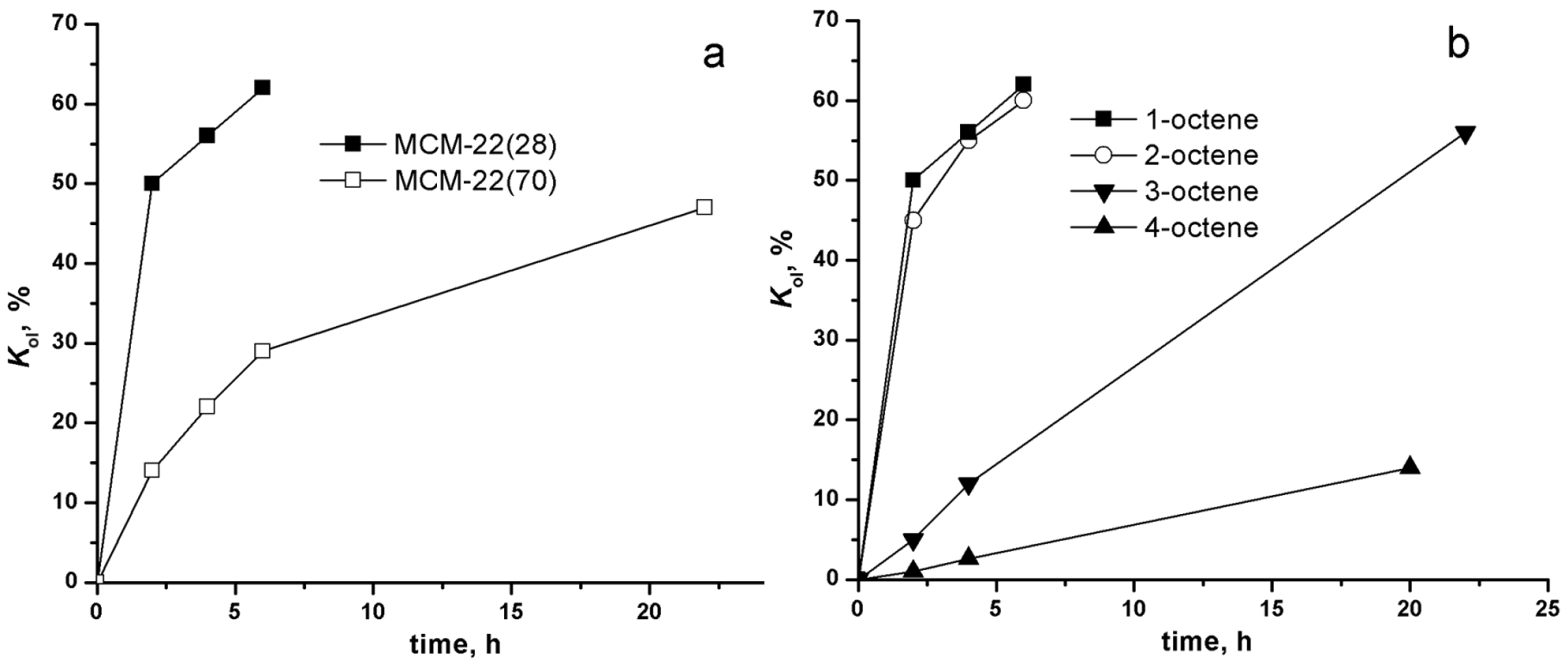
Conversion to oligomers for 1-octene over MCM-22(28) and MCM-22(70) (a) and conversion to oligomers for 1-octene, 2-octene, 3-octene, and 4-octene over MCM-22(28) (b). 50 mg Support, 1.5 mL octene, 40 °C.

The experiments with Mo-free zeolites (Figure 5a,b) confirmed that the oligomerization activity was connected with the support itself. In these “blank” experiments the reaction conditions, as well as pretreatment mode were the same as for Mo oxide catalysts. No metathesis products were observed, only 1-octene oligomerization and double bond isomerization occurred. Figure 5a shows 1-octene oligomerization over MCM-22(28) and MCM-22(70). Families of dimers and trimers (in weight ratio dimers/trimers approximately 20:1 for the final conversions) were detected, isolation and characterization of individual dimers/trimer was not possible. It was visible from GC, that isomerization of starting 1-octene also occurred, however, the exact quantification was not possible. The oligomerization rate was higher for MCM-22(28) in accord with its higher acidity as compared with MCM-22(70). The extent of oligomerization in these blank experiments is several times higher than that achieved over metathesis catalysts: it may be due to the partial capping of support acid sites with Mo species catalysts and also due the parallel consumption of 1-octene in metathesis.

Figure 5b shows oligomerization of 1-octene, 2-octene (*cis* + *trans*), 3-octene (*trans*), and 4-octene (*trans*) over MCM-22(28). It is seen that the initial reaction rate decreases in the order 1-octene ≈ 2-octene > 3-octene > 4-octene. The low-temperature oligomerization of alkenes over zeolite was studied as concerns industrially important low alkenes oligomerization and lower reactivity of internal alkenes in comparison with 1-alkenes was also recognized [30–31]. The reduced activity of 3- and 4-octenes in oligomerization might explain the fact, that in our metathesis experiments the accompanying oligomerization occurred practically only in the beginning of the reaction. In later stages when most of 1-octene was isomerized to 3- and 4-octenes only little increase in oligomer amounts was observed.

## Conclusion

3D and 2D zeolites of MWW (MCM-22 and MCM-56) and MFI topologies were used for the first time as supports for the preparation of highly active molybdenum oxide metathesis catalysts. The catalysts, prepared by thermal spreading of MoO_3_ and/or MoO_2_(acac)_2_ on these supports in NH_4_^+^ forms (6 wt % and/or 5 wt % of Mo) were tested in neat 1-octene metathesis under mild conditions (batch reactor, atmospheric pressure, 40 °C).

The catalyst activity (expressed as *K*_tot_ values at the reaction time = 2 h) decreased in the order 6MoO_3_/MCM-22(28) > 6MoO_3_/MCM-56(13) > 6MoO_3_/2D-MFI(26) > 6MoO_2_(acac)_2_/MCM-22(70) >> 6MoO_3_/HZSM-5(25). This activity order reflects two effects enhancing the activity: (i) support acidity and (ii) structure characteristics ensuring good accessibility of active species by substrate molecules. The most active 6MoO_3_/MCM-22(28) exhibited a significantly higher activity than that of a similar catalyst supported on siliceous mesoporous molecular sieve SBA-15.

Due to the catalyst acidity accompanying reactions occurred: (i) 1-octene double bond isomerization followed by cross metathesis and (ii) 1-octene oligomerization (mainly dimerization). The extent of these reactions depends strongly on the support acidity. Highly acidic supports MCM-22(28) and MCM-56(13) delivered a catalyst of rather low selectivity (up to 14% conversion to oligomers, 15–20% conversion to tetradecene at about *K*_tot_ = 90%). Less acidic supports – MCM-22(70) and 2D-MFI(26) gave rise catalysts of significantly higher selectivity: conversion to oligomers was reduced to 1%, double bond isomerization and cross metathesis proceeded in less extent, so selectivity to tetradecene increased (e.g., for 2D-MFI(26) to 35% at *K*_tot_ = 90%).

It is seen that for the metathesis of longer chain hydrocarbons like 1-octene, supports ensuring a good access of bulkier substrate to the active centers are necessary. The acidity of the support increases the catalyst activity, however, simultaneously with decrease of the catalyst selectivity. 2D-MFI(26) due to its moderate acidity and 2D character results in catalysts of moderate activity but of the highest selectivity.

With the described catalysts 1-octene was converted into a mixture of higher olefins: in addition to tetradecene as a homometathesis product, olefins of 9–13 C atoms from cross metathesis and C16 dimers were formed in various extent. Therefore, the described catalysts may find application especially if a mixture of higher olefins is desired, for example in the preparation of detergents, lubricants etc.

## Experimental

### Catalyst preparation and characterization

The zeolite supports MCM-22 and MCM-56 were prepared according to [32–33], 2D-MFI was synthesized according to [21]. HZSM-5 (CBV 5524) was purchased from Zeolyst. Na^+^ forms of zeolites were converted to NH_4_^+^ form by three-fold treatment with 1.0 M NH_4_NO_3_ solution at room temperature for 3 h. The supports were characterized by XRD (Bruker AXS D8 Advance diffractometer with a graphite monochromator and a Vantec-1 position sensitive detector using Cu Kα radiation in Bragg−Brentano geometry) and by N_2_ adsorption (77 K, Micromeritics GEMINI II 2370 volumetric Surface Area Analyzer). Molybdenum(VI) oxide (Sigma-Aldrich) and bis(acetylacetonato)dioxomolybdenum(VI) (Aldrich) as sources of Mo oxide species were used for catalyst preparation using the thermal spreading method (500 °C, 8 h). SEM images were recorded using a JEOL JSM-5500LV microscope.

The concentrations of Lewis (*c*L) and Brønsted (*c*B) acid sites were determined by FTIR spectroscopy of adsorbed pyridine (Py) using a Nicolet 6700 with a transmission MCT/B detector. The zeolites were pressed into self-supporting wafers with a density of 8.0–12 mg·cm^–2^ and activated in situ at *T* = 450 °C and *p* = 5·10^–5^ torr for 4 h. Pyridine adsorption was carried out at 150 °C and a partial pressure of 3.5 torr for 20 min followed by desorption for 20 min at 150, 250, 350 or 450 °C. Before adsorption, pyridine was degassed by freeze–pump–thaw cycles. All spectra were recorded with a resolution of 4 cm^–1^ by collecting 128 scans for a single spectrum at room temperature. The spectra were recalculated using a wafer density of 10 mg·cm^–2^. *c*L and *c*B were evaluated from the integral intensities of bands at 1454 cm^–1^ (*c*L) and 1545 cm^–1^ (*c*B) using extinction coefficients, ε(L)=2.22 cm·mmol^–1^ and ε (B)=1.67 cm·mmol^–1^[34].

For elemental analysis ICP OES (iCAP 7000, Thermo Scientific) was used. About 50 mg of the catalyst was digested in a mixture of HF, HCl, and HNO_3_ (1:2:2). The samples were placed in a Berghof microwave in a closed vessel at *T* = 140 °C for 35 min. Saturated solution of H_3_BO_3_ was then added for complexation of the excess of HF. After digestion solutions under analysis were collected in 250 mL flasks and diluted with ultra pure water.

### Catalytic experiments

Catalytic experiments were carried out in an argon atmosphere using a vacuum argon line. 1-Octene (Aldrich, 98%) was passed through alumina and stored with Na. The content of water in 1-octene was about 5 ppm. 2-Octene (Alfa-Aesar, 98%), *trans*-3-octene (Alfa-Aesar, 97%) and *trans*-4-octene (Aldrich) were purified in a similar way. In a typical experiment 50 mg of catalyst (6 wt % of Mo) was used. Before reaction catalyst was pretreated in vacuo at 500 °C for 30 min. After cooling to 40 °C, the reactor was filled with Ar and neat 1-octene (1-octene/Mo ratio = 320) was added under stirring. The reaction progress was followed by GC analysis of reaction mixture samples taken at given intervals. Individual compounds were identified by GC/MS. A high-resolution gas chromatograph Agilent 6890 with a DB-5 column (length: 50 m, inner diameter: 320 μm, stationary phase thickness: 1 μm), equipped with a 7683 Automatic Liquid Sampler and a FID detector and GC/MS (ThermoFinnigan, FOCUS DSQ II single Quadrupole) were used. Conversions were calculated from the mass balance.

## Supporting Information

File 1 IR spectra of catalysts, GC of reaction products, and SEM images of catalysts.
