# Supplementary material for: MoO3 on zeolites MCM-22, MCM-56 and 2D-MFI as catalysts for 1-octene metathesis
Source: Beilstein J Org Chem. 2018 Nov 27;14:2931–9. doi: 10.3762/bjoc.14.272 (PMC6278768; doi:10.3762/bjoc.14.272)
Supplement: File 1 — IR spectra of catalysts, GC of reaction products, and SEM images of catalysts. [file Beilstein_J_Org_Chem-14-2931-s001.pdf]

BEILSTEIN JOURNAL OF ORGANIC CHEMISTRY

Supporting Information

for

MoO_3 on zeolites MCM-22, MCM-56 and 2D-MFI as catalysts for 1-octene metathesis

Hynek Balcar, Martin Kubů, Naděžda Žilková and Mariya Shamzhy

Beilstein J. Org. Chem. **2018,** *14,* 2931–2939. doi:10.3762/bjoc.14.272

IR spectra of catalysts, GC of reaction products, and SEM images of catalysts

License and Terms: This is a supporting information file under the terms of the Creative Commons Attribution License (http://creativecommons.org/licenses/by/4.0). Please note that the reuse, redistribution and reproduction in particular requires that the authors and source are credited.
The license is subject to the *Beilstein Journal of Organic Chemistry* terms and conditions: (https://www.beilstein-journals.org/bjoc)

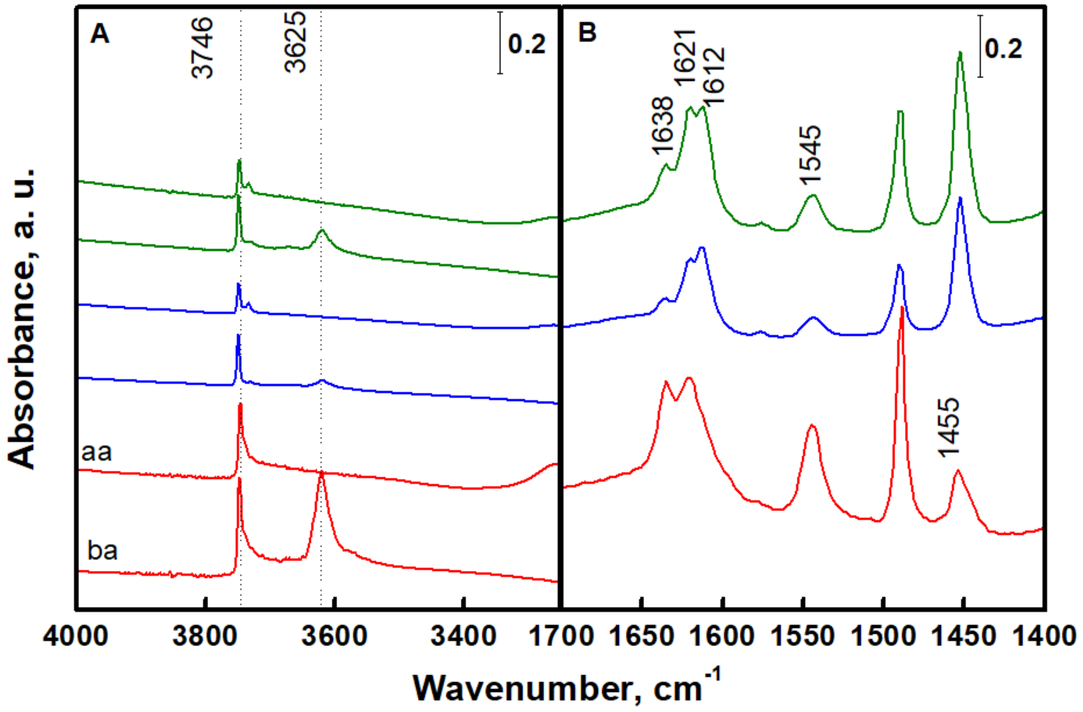

Figure S1: IR spectra of MCM-22(28), $6MoO_3$/MCM-22(28) and $6MoO_2(acac)_2$/MCM-22(28). A- region of hydroxy vibrations, B- region of pyridine vibrations. Bottom spectra – before pyridine adsorption, top spectra – after pyridine absorption.
Bands in the region 1445–1455 cm^{-1} correspond to Lewis acid sites, band at 1545 cm^{-1} to Brønsted acid sites.

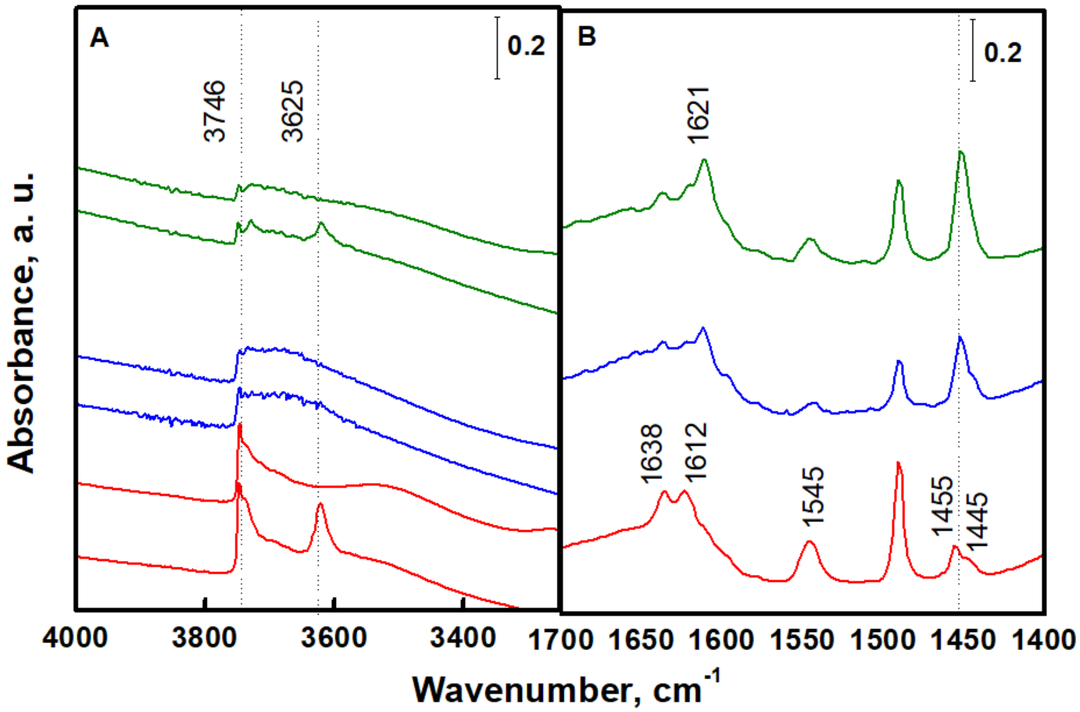

Figure S2: IR spectra of MCM-22(70), $6MoO_3$/MCM-22(70) and $6MoO_2(acac)_2$/MCM-22(70). A- region of hydroxy vibrations, B- region of pyridine vibrations. Bottom spectra – before pyridine adsorption, top spectra – after pyridine absorption.

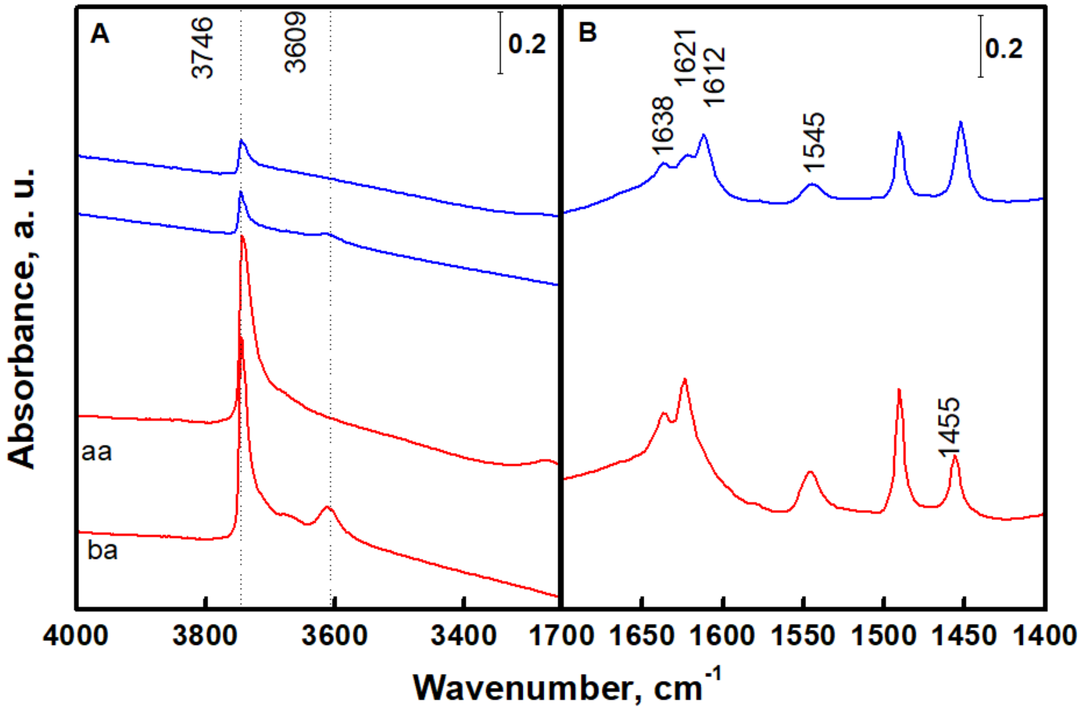

Figure S3: IR spectra of 2D-MFI(26) and $6MoO_3$/2D-MFI (26). A- region of hydroxy vibrations, B- region of pyridine vibrations. Bottom spectra – before pyridine adsorption, top spectra – after pyridine absorption.

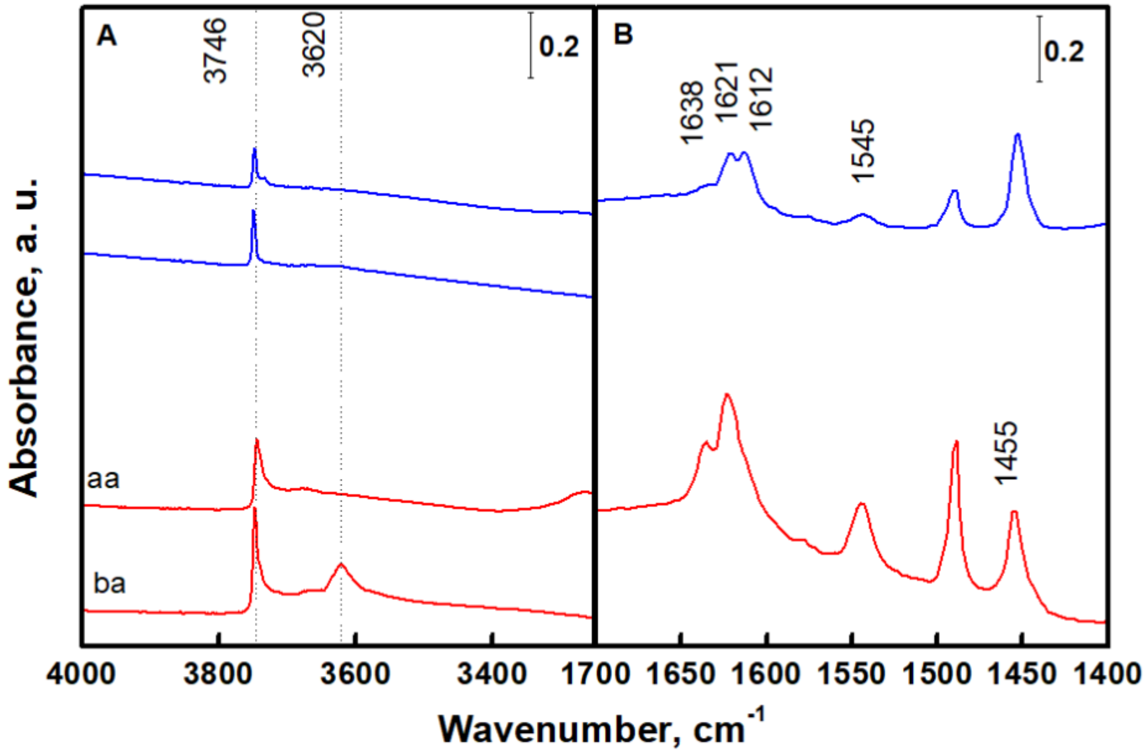

Figure S4: IR spectra of MCM-56(13) and 6MoO_3/MCM-56(13). A- region of hydroxy vibrations, B- region of pyridine vibrations. Bottom spectra – before pyridine adsorption, top spectra – after pyridine absorption.

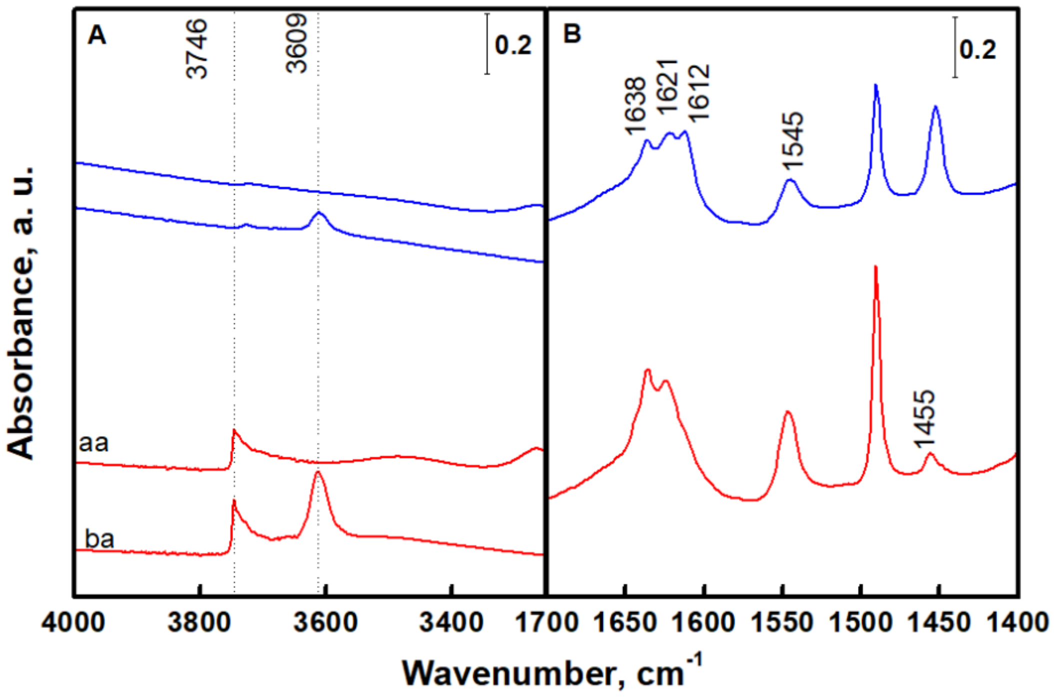

Figure S5: IR spectra of ZSM-5(25) and $6MoO_3$/ZSM-5(25). A- region of hydroxy vibrations, B- region of pyridine vibrations. Bottom spectra – before pyridine adsorption, top spectra – after pyridine absorption.

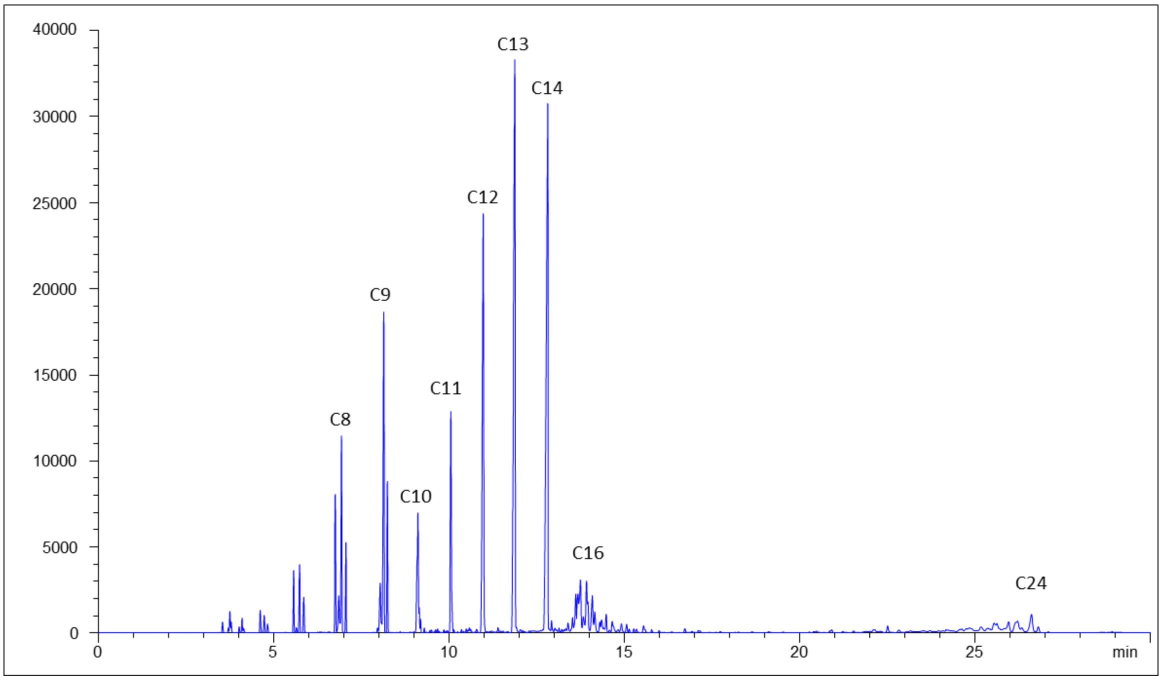

Figure. S6: Metathesis of neat 1-octene over $6MoO_3$/MCM-22(28) – GC of final products.

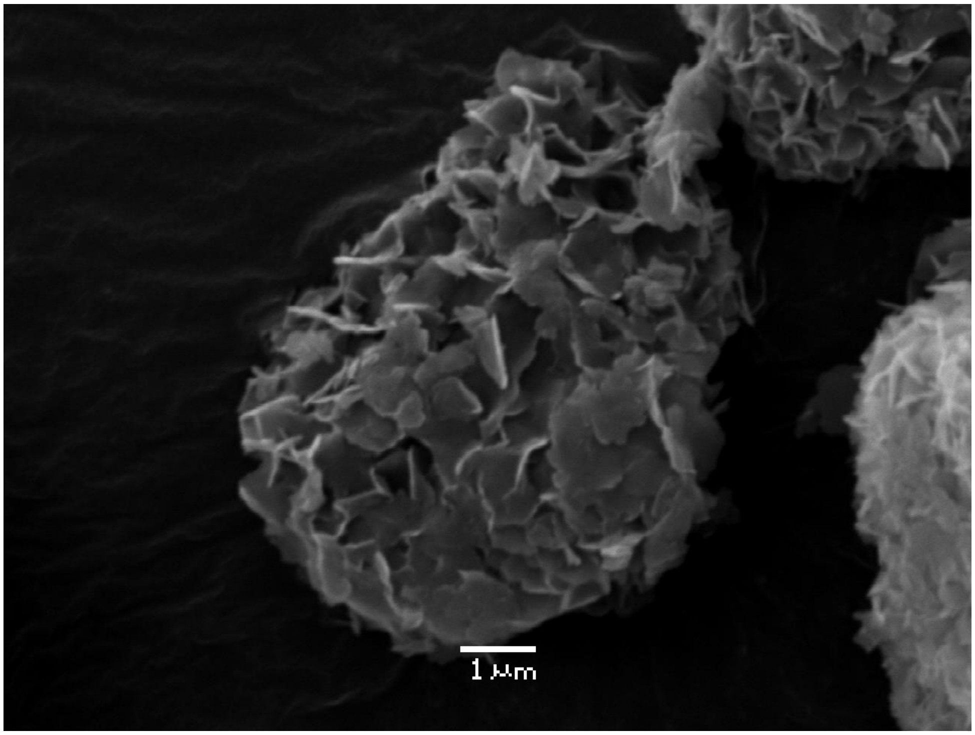

Figure S7: SEM image of MCM-22(28).

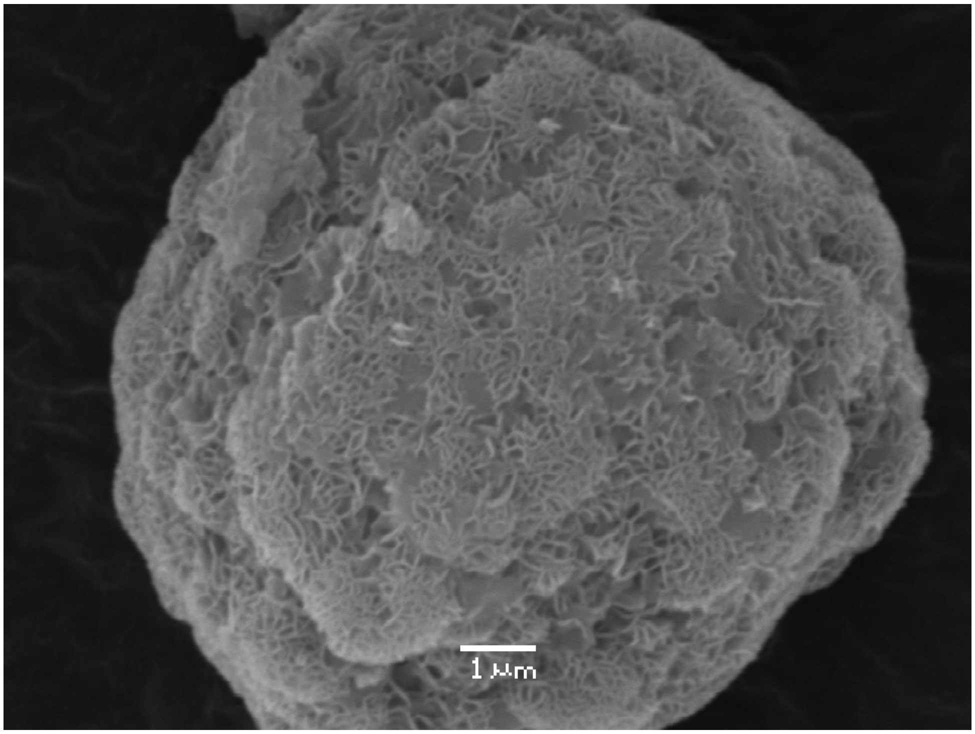

Figure S8: SEM image of MCM-58(13).
